# Transcriptional analysis of genes involved in nodulation in soybean roots inoculated with *Bradyrhizobium japonicum* strain CPAC 15

**DOI:** 10.1186/1471-2164-14-153

**Published:** 2013-03-06

**Authors:** Gesiele Almeida Barros de Carvalho, Jesiane Stefânia Silva Batista, Francismar Corrêa Marcelino-Guimarães, Leandro Costa do Nascimento, Mariangela Hungria

**Affiliations:** 1 Empresa Brasileira de Pesquisa Agropecuária, CNPSo, PO Box 231, Londrina, Paraná 86001-970, Brazil; 2 Department of Biochemistry and Biotechnology, Universidade Estadual de Londrina, PO Box 6001, Londrina, Paraná 86051-990, Brazil; 3 Laboratório de Genômica e Expressão, Institute of Biology, Universidade Estadual de Campinas, Rua Monteiro Lobato, 255, Campinas, São Paulo 13083-862, Brazil

**Keywords:** Subtractive library, Differential expression of genes, Nodulation

## Abstract

**Background:**

Biological nitrogen fixation in root nodules is a process of great importance to crops of soybean [*Glycine max* (L.) Merr.], as it may provide the bulk of the plant’s needs for nitrogen. Legume nodulation involves several complex steps and, although studied for many decades, much remains to be understood.

**Results:**

This research aimed at analyzing the global expression of genes in soybean roots of a Brazilian cultivar (Conquista) inoculated with *Bradyrhizobium japonicum* CPAC 15, a strain broadly used in commercial inoculants in Brazil. To achieve this, we used the suppressive subtractive hybridization (SSH) technique combined with Illumina sequencing. The subtractive library (non-inoculated x inoculated) of soybean roots resulted in 3,210 differentially expressed transcripts at 10 days after inoculation were studied. The data were grouped according to the ontologies of the molecular functions and biological processes. Several classes of genes were confirmed as related to N_2_ fixation and others were reported for the first time.

**Conclusions:**

During nodule formation, a higher percentage of genes were related to primary metabolism, cell-wall modifications and the antioxidant defense system. Putative symbiotic functions were attributed to some of these genes for the first time.

## Background

Soybean (*Glycine max* (L.) Merr.) has significant agronomic and nutritional relevance because of the high concentrations of protein and oil in its grains. Concomitant with the high protein content, the legume shows a strong demand for nitrogen (N) for optimal development and grain productivity [1]. Although atmospheric N_2_ is abundant, no eukaryotic organism is able to directly assimilate it, due to the strong triple bond linking the atoms [2,3]. However, when growing in N-depleted soils, much of soybean’s need for N can be obtained via biological nitrogen fixation (BNF) in root nodules, through the symbiotic association with bacteria, collectively called rhizobia, belonging mainly to the species *Bradyrhizobium japonicum* and *Bradyrhizobium elkanii*[4,5].

In Brazil, estimates indicate that the value of BNF by soybean root nodules is equivalent to U$7 billion/year; furthermore, BNF is an environmentally beneficial process, in comparison to the use of synthetic N fertilizers [4,5].

The establishment of the symbiosis starts with molecular interactions between the rhizobia and the host plant, involving a succession of complex processes that lead to profound changes in both symbionts [3,6]. The plant releases molecular signals, in particular flavonoid compounds, that are primary inducers of rhizobial nodulation genes. The induction of this class of genes leads to the biosynthesis of Nod factors, rhizobial signals that trigger specific responses in the root hairs of the host plant; plant cells perceive the presence of Nod factors—and of the rhizobia—through cell-surface receptors on the roots [7-10]. Many molecular events are triggered in a coordinated manner, leading to morphological and physiological changes in the host plant, necessary for a successful symbiosis [3].

Given the complexity of the symbiosis, studies of transcriptional profiles during nodulation are important to gain greater understanding of the nodulation process. Studies conducted with soybean, e.g. Brechenmacher et al. [2], evaluated gene-expression profiles in roots inoculated with *B. japonicum* (USDA 110), and elucidated reduced plant defenses. Furthermore, a complex regulatory mechanism in the plant was detected, enabling it to adapt to changes in its nutritional status. Another study of the transcriptome of soybean roots inoculated with strain USDA 110 showed rapid changes in gene expression in response to inoculation, with the pattern modified in accordance with the various stages of nodule development and function [11].

Brazil is the second most prolific producer of soybean worldwide, and probably the country where BNF has been exploited most successfully [4,5]. However, no investigation of transcriptomics with Brazilian strains and cultivars have been reported. This study aimed at analyzing the global expression of genes in soybean roots of cultivar Conquista inoculated with *B. japonicum* strain CPAC 15 (=SEMIA 5079), both broadly used in Brazil, through the suppressive subtractive hybridization (SSH) technique [12] combined with sequencing Illumina analysis.

## Results and discussion

The SSH library of the Brazilian cultivar Conquista, at 10 days after inoculation (DAI) with *B. japonicum* strain CPAC 15, which is broadly used in commercial inoculants in Brazil, resulted in 4,621,072 reads.

Among the 3,776 sequences identified as up-regulated, 3,210 were automatically recorded by the AutoFACT tool and grouped into functional categories (GO), according to the ontologies of molecular function (1,764 sequences) and biological process (1,728 sequences), using Blast2GO software [13]. The remaining 566 sequences presented no similarity with a known protein of the database.

**Figure 1:**
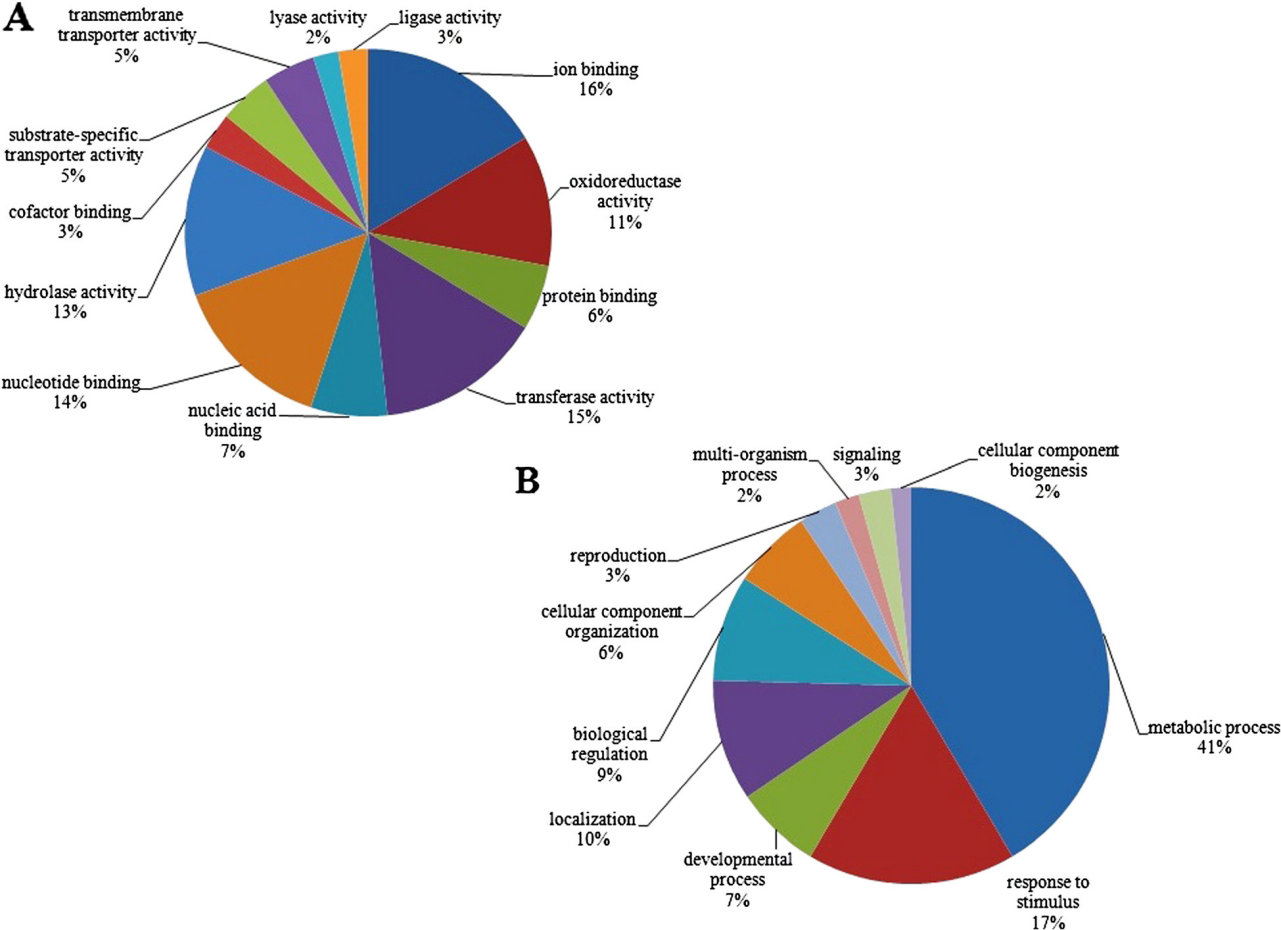
**Functional categorization of the transcripts obtained in the analysis of a suppressive subtractive hybridization library built with soybean roots mock-inoculated *versus *inoculated with *Bradyrhizobium japonicum *strain CPAC 15 (=SEMIA 5079).** Fraction of sequences (%) classified according to (**A**) molecular functions; and (**B**) biological processes.

Concerning molecular function (Figure 1A), the categories including more reads were those of ion binding, followed by transferase activity, nucleotide binding, hydrolase activity and oxidoreductase activity, demonstrating intense metabolic activity occurring during the nodulation process. Figure 1B highlights the biological processes triggered in the host plant, at 10 DAI with rhizobia, where the most representative category was of the metabolic process, followed by response to stimulus. Table 1 displays a detailed description of the main biological processes, triggered in the soybean roots by the presence of the nitrogen-fixing bacteria. In general, our results are consistent with those obtained with other legumes evaluated during nodulation, for example, *Medicago truncatula,* in which many physiological processes were increased by the presence of rhizobia, leading to the induction of genes involved in signaling, transcriptional regulation, intracellular calcium oscillations, oxidative explosion, cell proliferation and alterations in the cytoskeleton, all necessary for nodule development and function [14-16].

**Table 1:** **Classification of biological processes triggered in soybean roots (cv. Conquista) in response to inoculation with strain CPAC 15 of *B. japonicum***

**ID GO**	**Description**	**N**^**o **^**Seq.**
GO:0008150	**Biological process**	1,728
GO:0008152	**I- Metabolic process**	1,290
GO:0044237	Cellular metabolic process	964
GO:0044238	Primary metabolic process	756
GO:0019748	Secondary metabolic process	62
GO:0006807	Nitrogen compound metabolic process	269
GO:0009058	Biosynthetic process	373
GO:0009056	Catabolic process	195
GO:0044281	Small molecule metabolic process	324
GO:0043170	**I.a-** Macromolecule metabolic process	625
GO:0019538	Protein metabolic process	337
GO:0010467	Gene expression	69
GO:0050896	**II- Response to stimulus**	529
GO:0070887	Response to chemical stimulus	246
GO:0009628	Response to abiotic stimulus	162
GO:0009607	Response to biotic stimulus	56
GO:0006950	**II.a-** Response to stress	289
GO:0006952	Defense response	68
GO:0009719	**II.b-** Response to endogenous stimulus	113
GO:0009725	Response to hormone stimulus	106
GO:0032502	**III- Developmental process**	218
GO:0007275	Multicellular organismal development	124
GO:0048856	**III.a-** Anatomical structure development	114
GO:0048513	Organ development	73
GO:0051179	**IV- Localization**	308
GO:0051641	Cellular localization	56
GO:0033036	Macromolecule localization	60
GO:0051234	**IV.a-** Establishment of localization	306
GO:0006810	Transport	265
GO:0065007	**V- Biological regulation**	269
GO:0050789	Regulation of biological process	208
GO:0065008	Regulation of biological quality	80
GO:0071840	**VI- Cellular component organization or biogenesis**	203
GO:0071841	Cellular component organization or biogenesis at cellular level	136
GO:0016043	Cellular component organization	171
GO:0044085	**VII- Cellular component biogenesis**	51
GO:0000003	**VIII- Reproduction**	97
GO:0051704	**IX- Multi-organism process**	62
GO:0051707	Response to other organism	51
GO:0023052	**X- Signaling**	82
GO:0007165	Signal transduction	82

Furthermore, studies with *Lotus japonicus* indicate that genes involved in across-membrane transport, hormone metabolism, cell-wall modification and signal transduction were also induced by the presence of the bacteria [17,18].

Moreover, we also identified in our library nodulin-21 [19], nodulin-22 [20], nodulin-26 [21], nodulin-36 [22], among others, which are recognized as induced genes in the tested condition, giving further confidence in SSH data.

For a more focused discussion of our results, categories were selected based on the high level of gene expression (RPKM, reads per kilobase of exon per million mapped reads), in comparison to the RPKM values of genes validated by RT-qPCR (Additional file 1: Table S1). Therefore, we assume that other genes with greater values of RPKM are also up-regulated during soybean nodulation.

### Metabolic process

Previous studies of the effects of inoculation of soybean with *B. japonicum*, at various stages of nodulation, ranging from hours (12, 24 and 48), days (4, 8 and 16) and weeks (2, 5 and 10) [2,11,23] after inoculation have reported profound changes in plant metabolism, that varied with the growth stage analyzed [24]. Thus, our study is in accordance with Hayashi et al. [25] because the Nod factors perception cause metabolic changes, and here we can observed that the metabolic pathways most active in the presence of rhizobia were glycolysis and the Krebs cycle (Additional file 2: Figures S1 and S2). Indeed, for the development and functioning of nodules, it is necessary to allocate plant sources of C to the new organs [26,27]. Similarly, genes involved in the breakdown of sucrose, glycolysis and synthesis of amino acids were found to be differentially expressed in *L. japonicus*, consistent with these processes being accelerated during nodulation [28]. The glycolytic pathway and Krebs cycle are closely linked, representing the main ways of acquiring energy. Consistent with the N_2_-fixing symbiosis being an energy-demanding process [26,29,30], the expression of genes related to both pathways increased. Additional file 1: Table S2 presents the identified genes related to energetic pathways; of these, Glyma14g36850.1 (fructose-biphosphate aldolase) had the highest level of expression based on its RPKM value. This enzyme participates in the production of dicarboxylic acids for rhizobial and carbon skeletons for the assimilation of N by the plant [14]. According to Brechenmacher et al. [2], this induction effect occurs from the eighth day after inoculation.

Among the metabolic processes, nodulation also changes the redox state of root cells, and a major metabolic pathway activated by CPAC 15 was the synthesis of glutathione (Additional file 2: Figure S3), which is protective against reactive oxygen species (ROS) [31]. The levels of expression of the genes for glutathione peroxidase, gamma-glutamyl transferase and glutathione S-transferase were higher than for the other genes encoding enzymes in this pathway (Table 2).

**Table 2:** **Genes that encode the soybean enzymes that participate in the metabolism of glutathione in the presence of *B. japonicum *strain CPAC 15**

**EC number**	**Description**	**Gene**	**RPKM**^**a**^	**N**^**o **^**reads**
1.1.1.42	isocitrate dehydrogenase	Glyma14g39160.3	525.736	1,323
1.1.1.44	6-phosphogluconic dehydrogenase	Glyma08g28230.1	366.492	616
1.1.1.49	glucose-6-phosphate dehydrogenase	Glyma19g24250.1	393.621	477
1.11.1.9	glutathione peroxidase	Glyma05g37900.3	3,425.42	2,444
1.8.1.7	glutathione reductase	Glyma16g27210.1	590.971	658
2.3.2.2	gamma-glutamyltransferase	Glyma11g35950.1	1,631.78	1,058
2.5.1.18	glutathione S-transferase	Glyma01g04690.1	1,392.19	344
		Glyma06g20730.1	1,102.13	1,774
		Glyma07g16910.1	806.678	1,954
		Glyma02g02860.1	1,013.09	1,019
		Glyma08g41960.1	693.606	1,347
		Glyma12g28670.2^b^	583.947	985
6.3.2.2	gamma-glutamylcysteine synthetase	Glyma05g37850.3	326.118	410
6.3.2.3	glutathione synthase	Glyma19g42600.1	363.132	148

^a^ Reads per kilobase of exon per million mapped reads.

^b^ Identified in proteomics analysis.

As shown by Chang et al. [32], changes in redox state are observed at various stages of nodulation. They occurred during early symbiotic interactions in *Medicago sativa*[33] and *Phaseolus vulgaris*[34]. Transcripts present in the subtractive library, which participate of antioxidant defense system, are shown in Table 2. In *Medicago truncatula,* inhibition of the synthesis of glutathione resulted in fewer nodules on inoculated roots, showing the critical role of glutathione in nodule morphogenesis [31,35].

### Signaling

Signal-transduction genes are important at the various stages of the symbiotic interaction, as they lead to the coordinated development of epidermal and cortical cells needed to permit rhizobial penetration and nodule initiation [8,36]. It is known that the processes of recognition and signaling triggered by the rhizobia activate genes related to nodulation; some of them have been characterized in soybean, such a receptor kinases NARK (nodule auto regulation receptor kinase) [37] and NORK (nodulation receptor kinase) [38], protein kinase type LRR (leucine rich repeat) encoded, respectively, by genes Glyma12g04390 and Glyma09g33510. Libault et al. [11] identified other genes related to the LRR proteins that are differentially regulated during nodulation.

Among the genes with high expression level identified in our library, two encoded LRRs (Table 3), highlighting Glyma16g29220.1, with the highest level of expression (based on RPKM values) among all genes of the SSH library. Therefore, this gene represents a novelty that may be an important receptor. Besides soybean, LRRs have also been identified in the roots of *Lotus japonicus*[39], *Medicago truncatula* and *Sesbania rostrata* inoculated with their specific symbionts [40].

**Table 3:** **Genes selected, based on RPKM, from some biological processes induced in soybean roots inoculated with strain CPAC 15 of *B. japonicum***

**Biological process**	**Genes**	**Description**	**RPKM**^**a**^	**N**^**o **^**reads**
Signaling	Glyma16g29220.1	LRR (*Leucine rich repeat*)	210,781	158,479
	Glyma03g32700.2	LRR (*Leucine rich repeat*)	1,943.56	1,149
	Glyma13g03910.1	Calmodulin	2,424.67	1,940
	Glyma05g13900.1	Calmodulin	1,743	2,510
	Glyma20g10820.1	Calmodulin	1,470.5	2,522
Response to stimulus	Glyma20g23080.1	Calreticulin	1,823.09	5,531
	Glyma15g02220.1	PDR (*Pleiotropic drug resistance*)	1,735.93	384
	Glyma08g29090.1	Glycoside hydrolase 19	1,177.12	1,319
	Glyma18g51980.1	Glycoside hydrolase 19	1,027.26	1,122
Transport	Glyma20g32000.1	Aquaporin	1,377.3	3,533
	Glyma10g35520.2	Aquaporin	1,613.69	3,245
	Glyma14g06680.5	Aquaporin	2,989.98	9,195
	Glyma12g29510.2	Aquaporin	1,744.05	4,347
	Glyma12g08040.1	Aquaporin	5,022.48	5,497
	Glyma05g32620.1	ABC transport	1,834.5	531
	Glyma08g00280.1	ABC transport	1,053.41	949
	Glyma08g28190.1	Amino acid transporter	1,734.53	4,473
	Glyma18g51220.1	Amino acid transporter	2,511.83	5,376
	Glyma20g33000.1	Amino acid transporter	1,273.41	1,325
	Glyma10g32750.1	Oligopeptide transporter	2,462.59	5,684
	Glyma19g45260.1	Potassium transporter	6,052.79	4,615
		Potassium transporter	1,865.16	1,468
		Potassium transporter	1,636.9	2,268
		Potassium transporter	2,088.8	3,890
	Glyma03g31310.1	Sulfate transporter	1,421.28	1,314

^a^ Reads per kilobase of exon per million mapped reads.

Also noteworthy are genes encoding calmodulins (Table 3), important proteins that participate in the transduction of signals triggered by the interaction of Nod factors to specific receptors on the surface of the root and, thus, allowing the expression of nodulins [41]. The most common calmodulin genes identified in the nodulation of soybean are Glyma15g35070.1 and Glyma08g24360.1 [42], and here three other genes were detected (encoding Glyma05g13900.1, Glyma13g03910.1, Glyma20g10820.1), whose participation in nodulation had not been emphasized.

### Response to stimulus

The category that combined the second largest number of transcripts was that of response to stimulus, which includes genes related to abiotic and biotic factors, including stress and defense responses and reactions to endogenous stimuli. These genes are involved in morphological, biochemical and physiological changes stimulated by inoculation with *B. japonicum.* Among the identified genes in this category we emphasize the calreticulin, protein PDR and glycoside hydrolases 19 (Table 3).

The calreticulins have great functional diversity; they participate in protein folding (chaperones), in the homeostasis of intracellular Ca^2+^ and signaling, and are also crucial for the growth and development of plants. Moreover, they are potent regulators of the plant in response to various stresses, including the activation of mechanisms of resistance to infections by pathogens [43-45]. These proteins have been broadly studied in animals, but their characterization in plant models remains limited [44]. Among the identified genes corresponding to calreticulins, Glyma20g23080.1 showed a high level of expression. Interestingly, no studies of the activities of these proteins during the nodulation have been reported.

Another gene detected in our study was related to a PDR-like-ABC transporter-family protein, involved in the responses to biotic and abiotic stresses in plants and fungi [46]. These proteins have been identified as transporters involved in the secretion of genistein in soybean roots in response to *B. japonicum*[47], thus with an important role in nodulation.

Genes encoding glycoside hydrolases-19 were also detected. They are also known as chitinases and, among other functions in the symbiosis, it is believed that they participate in the perception and degradation of Nod factors [48,49]. Xie et al. [50] showed an increase in activity of chitinase in the presence of Nod factors of rhizobia in soybean roots, suggesting that these enzymes can regulate the perception of Nod factors during nodulation.

### Transport

Genes related to transport accounted for about 10% of differentially expressed genes; Table 3 displays the genes with the highest expression levels. This category was also expressed in the nodulation of *M. truncatula*[51] and includes, among others, genes encoding membrane-transport proteins. Aquaporins, membrane proteins responsible for transport, especially of water, are expressed [52-54]. The soybean genes encoding aquaporins most commonly found on nodulation are Nodulin-26 (Glyma08g12650, Glyma-19g22210) found in the symbiosome [11,42]. Other genes encoding aquaporins were identified in our study (Table 3). During nodulation in *Medicago truncatula*, the aquaporins were the most expressed membrane proteins from early to relatively late stages of nodulation [14], emphasizing their importance during nodule organogenesis.

Other transporters are important for the acquisition of nutrients by root cells and the symbiosome [55]. Expression was detected of genes related to ABC transport, amino acid transport, and oligopeptide, potassium and sulfate transport (Table 3).

### Cell-wall modification

Given the need for structural modification of the root during infection by rhizobia, several genes are involved in plant cell-wall penetration and cytoskeletal reorganization. Some genes involved in cell-wall modification encode enzymes involved in carbohydrate metabolism (Additional file 2: Figure S4). This is particularly important in nodulation, because the transcripts that encode enzymes (Table 4) active in this pathway may be acting specifically on the reorganization of the root and the formation of nodular structure.

**Table 4:** Genes encoding soybean enzymes active involved in carbohydrate metabolism

**EC number**	**Description**	**Gene**	**RPKM**^**a**^	**N**^**o **^**reads**
1.1.1.22	UDP-glucose 6-dehydrogenase	Glyma01g06970.1	1,118.45	2,934
2.4.1.12	cellulose synthase	Glyma05g32100.1	1,225.43	1,688
2.4.1.13	sucrose synthase	Glyma15g20180.3^b^	1,552.25	5,024
2.4.1.25	4-alpha-glucanotransferase	Glyma03g27600.1	160.692	105
2.4.1.34	callose synthetase	Glyma06g44770.1	441.939	201
2.7.1.1	Hexokinase	Glyma11g01820.1	118.426	34
3.1.1.11	pectinesterase	Glyma03g03460.1	2,433.21	2,497
3.2.1.15	polygalacturonase	Glyma05g37490.1	761.534	1,405
3.2.1.2	beta-amylase	Glyma06g45700.1	189.028	391
3.2.1.26	invertase	Glyma20g31730.1	677.598	1,994
3.2.1.39	glucan endo-1,3-beta-D-glucosidase	Glyma03g28850.1	2,612.62	6,736
3.2.1.4	cellulase	Glyma05g36930.1	608.64	417
4.1.1.35	UDP-glucuronate decarboxylase	Glyma12g06980.3	193.024	268
5.4.2.2	phosphoglucomutase	Glyma05g34790.1	1,159.25	2,329

^a^ Reads per kilobase of exon per million mapped reads.

^b^ Identified in proteomics analysis.

A study by Kaewsuralikhit et al. [56], of soybean at 12 DAI, showed elevated expression of pectinesterase, one of the enzymes responsible for cell-wall degradation during the formation of nodules, which also occurs in *Sesbania rostrata*[57]. In *Medicago truncatula*, a pectinesterase was up-regulated and cellulase was induced on the third and fourth days post-inoculation [14]. And in the present study, we also identified the gene that encode pectinesterase 10 DAI, confirming that this gene is induced only some days post-inoculation, because in the early hours, the pectinesterase gene showed as down-regulated [25].

Another important enzyme with a high level of expression in this study was sucrose synthase (Nodulin-100), which, among other known functions in nodulation, contributes significantly to development of cell wall [58].

### SSH validation by RT-qPCR and proteomics

Two genes, represented by Glyma16g06940 and Glyma18g05710, were chosen for SSH validation by RT-qPCR. We took the genes that showed RPKM values of 460.98 and 397.18 respectively, in order to verify the sensitivity and quality of the subtraction. Expression levels of both genes were up-regulated at the same time, 10 DAI (Additional file 1: Table S1).

Proteomics was used as a supplemental functional analysis, in view to validate the transcriptional data. In parallel with the RNA extraction, we also made the protein extraction of both conditions (inoculated and non-inoculated). Two-dimensional gel electrophoresis profiles of the two conditions were compared with each other. Representative spots, which showed a significant higher volume in the inoculated condition, were selected and identified by mass spectrometry.

Two spots were successfully identified and one of the selected spots didn’t fit into the statistical parameters of identification. These two proteins identified were also found in the subtractive library data, which represents a functional confirmation of the transcriptomic analysis. Two of them were only detected in the inoculated condition: a putative glutathione-S-transferase (Glyma12g28670.2). The other identified protein was a sucrose synthase (Glyma15g20180.3), which had a 1.47 fold change in relation to the non-inoculated condition (Additional file 2: Figure S5). It is also worth mentioning that both spots identified correspond to the same Glyma IDs identified by the RNAseq analysis.

## Conclusions

By using the SSH technique, it was possible to identify the main biological processes triggered in the Brazilian soybean cultivar Conquista after inoculation with the commercial strain CPAC 15 of *B. japonicum*. Among the main processes, we may highlight the metabolic pathways of primary metabolism, cell-wall modification and antioxidant-defense systems. Putative functions for some of these genes were assigned for the first time in the *Bradyrhizobium*-soybean symbiosis.

The analysis of transcriptional profiles of soybean in the presence of *B. japonicum* is essential to understand the symbiosis. Some transcripts have been previously described in nodulated soybean; however, novel genes were firstly described and could be related to the Brazilian germoplasm (plant genotype and bacterium), both studied by this standpoint for the first time.

## Methods

### Plant material

Soybean seeds of cultivar Conquista MG/BR46 were surface sterilized [59] and germinated on absorbent paper moistened with sterile distilled water, at 22 ± 2°C (in the dark) for three days, and seedlings were transferred to sterile plastic bags containing 200 mL of N-free nutrient solution [60].

### Inoculum preparation and plant inoculation

The *B. japonicum* strain used for inoculation was CPAC 15 (=SEMIA 5079), which has been used in commercial inoculants in Brazil, since 1992, for its outstanding symbiotic efficiency and competitiveness [4,5]. The bacterium was grown until the exponential phase of growth in yeast-mannitol broth (YMB) [59]. The cells were centrifuged and washed with saline solution (NaCl 0.85%). Aliquots of washed cell suspension were counted in YMB medium, indicating a concentration of 2.27 × 10^7^ cells mL^–1^.

The experiment had a fully randomized design with three replicates, each consisting of 20 plants per treatment. Treatments consisted of: soybean roots inoculated with strain CPAC 15 and non-inoculated soybean. For the inoculated treatment, 1 mL of the inoculum was applied at the base of each radicle. The plants were grown under greenhouse condition with a 12-h day/night period and mean temperature of 25–28°C/15–18°C (day/night) for ten days. Subsequently, the roots were separated from shoots, immediately frozen in liquid nitrogen and stored at −80°C.

### RNA extraction and isolation of mRNA

Total RNA was isolated from the roots of each treatment using Trizol (Invitrogen, Carlsbad, CA, USA), according to the manufacturer’s instructions. After extraction, total RNA was analyzed for quality using the Thermo Scientific NanoDrop ND-1000 spectrophotometer (NanoDrop Technologies, Wilmington, DE, USA). The mRNA was obtained from 2 μg of total RNA using the FastTrack MAG mRNA Isolation Kit (Invitrogen), according to the manufacturer’s specifications.

### Construction of the suppressive subtractive hybridization (SSH) cDNA library

This study is a component of a consortium named Genosoja (Brazilian Soybean Genome Consortium). Before the development of all experiments of the Genosoja project, the techniques of RNA extraction, SSH, RNAseq and qPCR were validated in several laboratories, and a standard protocol was applied, including the bioinformatics analyses. The experimental validations were published in a special edition of the Journal “Genetics and Molecular Biology” (v.35, n.1, 2012, http://www.scielo.br/scielo.php?script=sci_issuetoc&pid=1415-475720120002&lng=en&nrm=iso). A procedure included in the validation was the comparison of SSH and superSAGE strategies, and resulted in congruent results. Due to the high costs we have decided to follow the SSH strategy.

The SSH library was constructed using a PCR-Select cDNA Subtration Kit (Clontech Laboratories Inc, Mountain View, CA, USA), according to the manufacturer’s instructions. A cDNA library was built, containing clones from the subtraction of inoculated plants *versus* non-inoculated plants. This subtractive hybridization was performed using sample cDNAs (*tester*) from inoculated plants, which was subtracted with cDNAs (*driver*) from non-inoculated plants. This kind of hybridization is called forward subtraction, to identify the induced genes (up-regulated).

### Sequencing and bioinformatics

The pool of cDNA resulting from the subtractive library was transferred directly to the sequencing reaction with the Genome Analyzer GAII technology Illumina, carried out by Fasteris S.A., in Switzerland.

The reads from sequencing were aligned against the GENOSOJA database (http://www.lge.ibi.unicamp.br/soybean) [61]. The generated sequences were assembled and preliminarily analyzed at the LGE (Laboratory of Genomic and Expression at UNICAMP, Campinas, São Paulo, Brazil). First, the reads from sequencing were aligned in the reference genome of soybean (Phytozome database) [42] using SOAP software [62], allowing a maximum of two mismatches.

In addition, a level of inference of gene expression by reads per kilobase of exon per million mapped reads (RPKM) was assigned, making it possible to infer the expression level of genes in the subtractive library [63].

AutoFACT software [64] was used for automatic annotation of the sequences, through several BLASTx searches (e-value cutoff of 1e-5) against protein databases including NR (non-redundant from NCBI), Swissprot [65] and KEGG [66]. Subsequently, the transcripts were subjected to functional categorization, which was performed using the Gene Ontology database (http://www.geneontology.org), with Blast2GO [13], enabling clustering of genes according to the biological processes ontology (level 2) and molecular function (level 3). The most relevant metabolic pathways (based on the KEGG database) were also identified.

### SSH validation by real-time qPCR analysis

Quantitative real-time PCR experiments were performed to validate the expression of two genes, whose RPKM values were relatively low, in order to compare other genes in the library. Therefore, a new plant-inoculation experiment was performed, under the conditions previously described. After extraction of total RNA, the samples were treated with deoxyribonuclease I, amplification grade (Invitrogen), according to the manufacturer’s instructions. The cDNA synthesis was carried out using a SuperScript III First-Strand Synthesis System for RT-PCR (Invitrogen), according to the manufacturer’s instructions.

Primers were designed using PrimerExpress 3.0 (Applied Biosystems, Foster City, CA, USA), and the sequences are available on Additional file 1: Table S3, along with the sequences of the primers for the reference genes for β-actin [67] and F-box [68], used as endogenous controls.

For the RT-PCR reactions, Platinum SYBR Green qPCR SuperMix-UDG (Invitrogen) was used. Each sample was run in triplicate along with the corresponding non-template controls containing water instead of cDNA. Amplification reactions were performed using a 7300 Real-Time PCR System thermal cycler (Applied Biosystems). The amplification cycles were as follows: 50°C for 2 min, 95°C for 10 min, 40 cycles at 95°C for 15 s, and 60°C for 1 min. For each sample, a threshold cycle (Ct) value was calculated based on the amplification curves by selecting the optimal ΔRn (emission of reporter dye over starting background fluorescence) in the exponential part of the amplification plot. The specificity of the amplified products was evaluated by dissociation-curve analyses. The relative linear amount of target molecules relative to the calibrator was calculated according to Pfaffl [69], with significant differences determined with the REST 2009 software (p < 0.05) (Relative Expression Software Tool [70]).

### Proteomics analysis

Whole-cell proteins of soybean roots were extracted, from both the inoculated and non-inoculated treatments, following the simplified method described by Rodrigues et al. [71]. IPG strips (pH 4–7, 13 cm, GE Healthcare, Uppsala, Sweden) were rehydrated overnight with aliquots of 350 μg of solubilized proteins. Next, the strips were submitted to isoelectric focalization and SDS-PAGE as described by Batista and Hungria [72]. Gels were stained overnight with Comassie Brilliant Blue (CBB) R-350 (GE Healthcare), destained in a solution of 40% ethanol and 10% acetic acid and scanned (ImageScanner LabScan v. 5.0).

Spots were strictly identified in the high-resolution digitalized gel images and analyzed by Image Master 2D Platinum v 5.0 software (GE Healthcare). Ratios of mean normalized spot volumes were calculated. All selected spots were manually confirmed and statistically evaluated (p < 0.05) upon Student’s **t**-test, using XLSTAT (Addinsoft, France, add-in to Microsoft Excel).

Spots which showed a significantly higher volume in the inoculated condition were excised and processed as described before [72], with trypsin (Gold, mass spectrometry grade, Promega, Madison, WI) at 37°C overnight. Tryptic peptides (0.5 μL) were mixed with saturated solution of HCCA (α-cyano-4-hydroxy-cinamic acid) in 50% acetonitrile, 0.1% TFA (trifluoroacetic acid). The mixture was spotted onto a MALDI sample plate and allowed to crystallize at room temperature. The same procedure was used for the standard peptide calibration mix (Bruker Daltonics).

For mass spectra acquisition, a MALDI-TOF-MS Autoflex spectrometer (Bruker Daltonics) was operated manually in the LIFT mode for MALDI-TOF/TOF, using the FlexControl 3.0 (Bruker Daltonics) software.

PMFs and MS/MS ions generated were searched against the public database NCBInr (National Center for Biotechnology Information non-redundant)/Viridiplantae, using the Mascot software v. 2.3 (Matrix Science). For protein searches, monoisotopic masses were used, considering a peptide tolerance of 150 ppm and allowance of one missed cleavage. When MS/MS was carried out, a tolerance of 0.3 Da was acceptable. Carbamidomethylation of cysteine and oxidation of methionine were considered fixed and variable modifications, respectively.

Identifications were only validated when the Mowse (molecular weight search) score was significant, above the recommended cutoff score. The spectrometry datasets are available at PRIDE (http://ebi.ac.uk/pride/) with the experiment accession number 14846 (Username: review03737, Password: +6Nx + E^Y).

## Competing interest

The authors declare that they have no scientific or financial competing interests.

## Author-recommended internet resource

Site for the data http://www.lge.ibi.unicamp.br/soybean. In Browse, subtractive libraries.

## Authors’ contributions

Conceived and designed the experiments: MH, FCM-G. Performed the experiments: GABdC, JSdSB. Analyzed the data: All authors. Contributed reagents/materials/analysis tools: MH Wrote the paper: All authors. All authors read and approved the final manuscript.

## Supplementary Material

Additional file 1: Table S1 Real- time PCR result of differential expression of two target genes from soybean roots inoculated (sample) and mock-inoculated (calibrator) using 2^-∆∆Ct^ method. **Table S2**. Genes which encode enzymes present in the glycolytic pathway (A) and in the Krebs cycle (B) found in the subtractive library of soybean cv. Conquista when inoculated with *B. japonicum* strain CPAC 15. **Table S3**- Sequences of the primers used in the RT-qPCR and sizes of the PCR products obtained. Click here for file

Additional file 2: Figure S1 Glycolysis pathway during nodulation of soybean cultivar Conquista at 10 days after inoculation with B. japonicum CPAC 15 (ID Kegg map00010). **Figure S2**. Krebs cycle of soybean cultivar Conquista at 10 days after inoculation with *B. japonicum* CPAC 15 (ID Kegg map00020). **Figure S3**. Glutathione metabolism – the antioxidant defense system present in the nodulation (ID Kegg- map00480). **Figure S4**. Carbohydrate metabolism involved in the reorganization of the plant cell wall during organogenesis of the nodule (ID Kegg-map00500). **Figure S5.** Spots of whole-cell protein extracts of soybean roots inoculated (left images) and non-inoculated (right images) with *B. japonicum*. Proteins identified corresponds to sucrose synthase (1) and putative glutathione-S-transferase (2). Click here for file
