# Supplementary material for: Transcriptional analysis of genes involved in nodulation in soybean roots inoculated with Bradyrhizobium japonicum strain CPAC 15
Source: BMC Genomics. 2013 Mar 6;14:153. doi: 10.1186/1471-2164-14-153 (PMC3608089; doi:10.1186/1471-2164-14-153)
Supplement: Additional file 1: Table S1 — Real- time PCR result of differential expression of two target genes from soybean roots inoculated (sample) and mock-inoculated (calibrator) using 2-∆∆Ct method. Table S2. Genes which encode enzymes present in the glycolytic pathway (A) and in the Krebs cycle (B) found in the subtractive library of soybean cv. Conquista when inoculated with B. japonicum strain CPAC 15. Table S3- Sequences of the primers used in the RT-qPCR and sizes of the PCR products obtained. [file 1471-2164-14-153-S1.doc]

**Additional file 1: Table S1**- Real- time PCR result of differential expression of two target genes from soybean roots inoculated (sample) and mock-inoculated (calibrator) using 2^-∆∆Ct^ method .

| **Gene** | **Fold change** | ***P* value** | | **^a^Result** | **^b^RPKM** |
| --- | --- | --- | --- | --- | --- |
| Leucine Rich Repeat (Glyma16g06940.1) | 1.193 | 0.048 | UP | | 460.98 |
|
| Leucine Rich Repeat (Glyma18g05710.1) | 1.348 | 0.041 | UP | | 397.18 |
|

β-actin (Stolf-Moreira et al. 2011) and F-box protein (Libault et al. 2008) were used as the reference genes for normalization.

**^a^**Reads per kilobase of exon per million mapped reads

**^b^**UP-regulated in sample group, in comparison to control group, (p<0.05)

**Additional file 1: Table S2** - Genes which encode enzymes present in the glycolytic pathway (A) and in the Krebs cycle (B) found in the subtractive library of soybean cv. Conquista when inoculated with *B. japonicum* strain CPAC 15.

| **A** | **EC number** | **Description** | **Gene** | ***RPKM** | **N. reads** |
| --- | --- | --- | --- | --- | --- |
|  | 1.1.1.1 | alcohol dehydrogenase | Glyma06g12780.3 | 1,582.95 | 3,624 |
|  | 1.2.1.3 | aldehyde dehydrogenase (NAD+) | Glyma18g18910.1 | 637.658 | 1,741 |
|  | 1.2.1.5 | aldehyde dehydrogenase [NAD(P)+] | Glyma04g42740.1 | 452.563 | 554 |
|  | 1.2.1.12 | glyceraldehyde-3-phosphate dehydrogenase | Glyma06g18110.6 | 706.073 | 2,376 |
|  | 1.2.4.1 | pyruvate dehydrogenase | Glyma14g36540.3 | 1,228.86 | 2,347 |
|  | 1.8.1.4 | dihydrolipoyl dehydrogenase | Glyma07g36040.1 | 1,059.63 | 442 |
|  | 2.7.1.1 | Hexokinase | Glyma11g01820.1 | 118.426 | 34 |
|  | 2.7.1.11 | 6-phosphofructokinase | Glyma20g01010.1 | 305.958 | 195 |
|  | 2.7.1.40 | pyruvate kinase | Glyma10g07480.2 | 539.465 | 751 |
|  | 2.7.2.3 | phosphoglycerate kinase | Glyma15g41550.1 | 1,145.85 | 3,074 |
|  | 3.1.3.11 | fructose 1,6-bisphosphatase | Glyma07g17180.1 | 162.054 | 90 |
|  | 4.1.1.1 | pyruvate decarboxylase | Glyma13g30490.1 | 241.564 | 616 |
|  | 4.1.1.49 | phosphoenolpyruvate carboxykinase | Glyma01g02330.1 | 365.184 | 421 |
|  | 4.1.2.13 | fructose-bisphosphate aldolase | Glyma14g36850.1 | 2,293.45 | 4,469 |
|  | 4.2.1.11 | Enolase | Glyma19g37520.1 | 1,027.5 | 3,759 |
|  | 5.3.1.1 | triose-phosphate isomerase | Glyma15g04290.2 | 1,544.91 | 1,812 |
|  | 5.4.2.2 | Phosphoglucomutase | Glyma05g34790.1 | 1,159.25 | 2,329 |
|  | 6.2.1.1 | acetyl-CoA synthetase | Glyma15g25170.1 | 1,174.42 | 655 |
| **B** | **EC number** | **Description** | **Gene** | **RPKM** | **N. reads** |
|  | 1.1.1.37 | malate dehydrogenase | Glyma10g00920.2 | 280.322 | 723 |
|  | 1.1.1.41 | isocitrate dehydrogenase (NAD+) | Glyma10g06590.2 | 240.644 | 217 |
|  | 1.1.1.42 | isocitrate dehydrogenase (NADP+) | Glyma14g39160.3 | 525.736 | 1,323 |
|  | 1.2.4.1 | pyruvate dehydrogenase | Glyma14g36540.3 | 1,228.86 | 2,347 |
|  | 1.8.1.4 | dihydrolipoyl dehydrogenase | Glyma07g36040.1 | 1,059.63 | 442 |
|  | 2.3.3.8 | ATP citrate lyase | Glyma09g04000.1 | 1,412.31 | 988 |
|  | 4.1.1.49 | phosphoenolpyruvate carboxykinase | Glyma01g02330.1 | 365.184 | 421 |
|  | 4.2.1.3 | aconitate hydratase | Glyma12g32000.1 | 971.002 | 1,523 |
|  | 6.2.1.5 | succinyl-CoA synthetase | Glyma09g29460.1 | 348.288 | 792 |

*Reads per kilobase of exon per million mapped reads

**Additional file 1: Table S3**- Sequences of the primers used in the RT-qPCR and sizes of the PCR products obtained.

| **Target Gene** | **Primers sequence** | **Amplicon size** |
| --- | --- | --- |
| Leucine Rich Repeat (Glyma16g06940.1) | **F**: 5'- CCACACCCAACAAGCCCTATT-3' | 114 bp |
|  | **R**: 5'- CTCCTTGGCAACCTGTTCCAT- 3' |  |
| Leucine Rich Repeat (Glyma18g05710.1) | **F**: 5'- CATATGGTCTACAATGCCAGAATCAG- 3' | 105 bp |
|  | **R**: 5'- GAGGCAGAAGATGAAGATGGTGTAC- 3' |  |
| **Reference Genes** | **Primers sequence** | **Amplicon size** |
| β-actin | **F:** 5' GAGCTATGAATTGCCTGATGG 3' | 118 bp |
|  | **R:** 5' CGTTTCATGAATTCCAGTAGC 3' |  |
| F-box protein family | **F**: 5'-AGATAGGGAAATGGTGCAGGT-3' | 93 bp |
|  | **R**: 5'-CTAATGGCAATTGCAGCTCTC-3' |  |
