# Supplementary material for: Transcriptional analysis of genes involved in nodulation in soybean roots inoculated with Bradyrhizobium japonicum strain CPAC 15
Source: BMC Genomics. 2013 Mar 6;14:153. doi: 10.1186/1471-2164-14-153 (PMC3608089; doi:10.1186/1471-2164-14-153)

**Additional file 2: Figure S1**- Glycolysis pathway during nodulation of soybean cultivar Conquista at 10 days after inoculation with *B. japonicum* CPAC 15 (ID Kegg map00010).

**
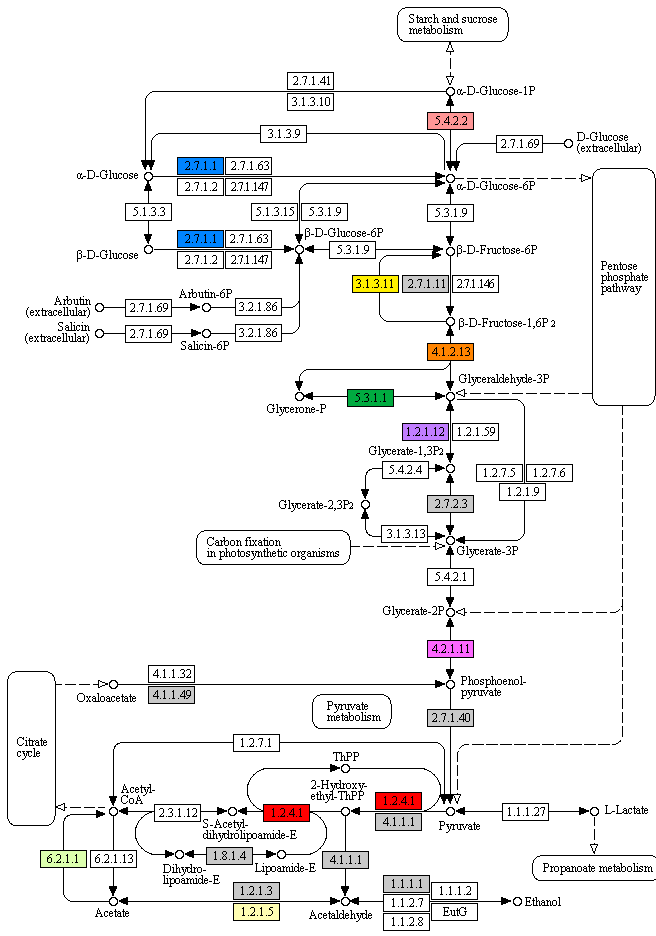
**

Note: The enzymes encoded by transcripts that are present in the subtractive library are highlighted with color combinations, in which the same color represents the same product.

**Additional file 2: Figure S2**- Krebs cycle of soybean cultivar Conquista at 10 days after inoculation with *B. japonicum* CPAC 15 (ID Kegg map00020).


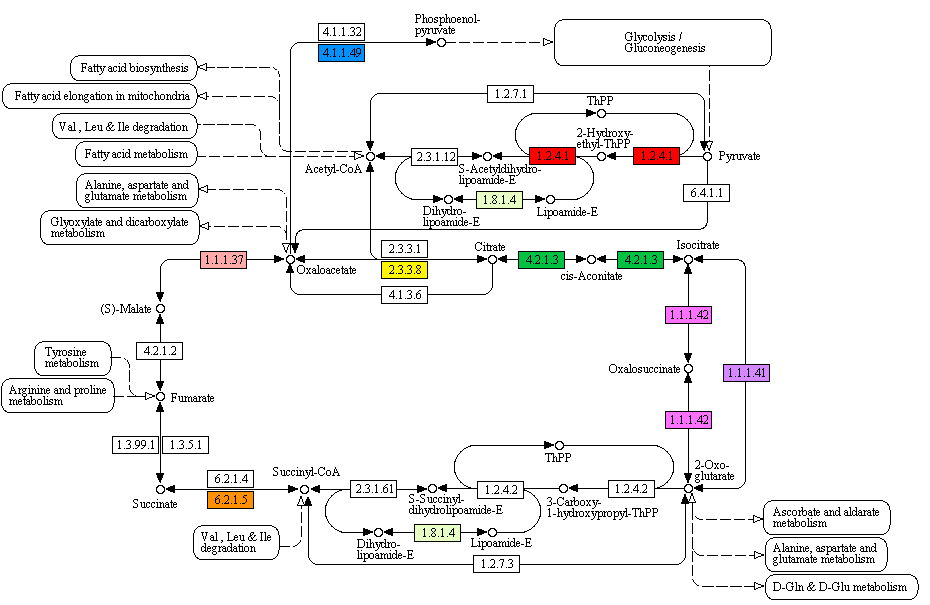


Note: The enzymes encoded by transcripts that are present in the subtractive library are highlighted with color combinations, in which the same color represents the same product.

**Additional file 2: Figure S3**- Glutathione metabolism – the antioxidant defense system present in the nodulation (ID Kegg- map00480).


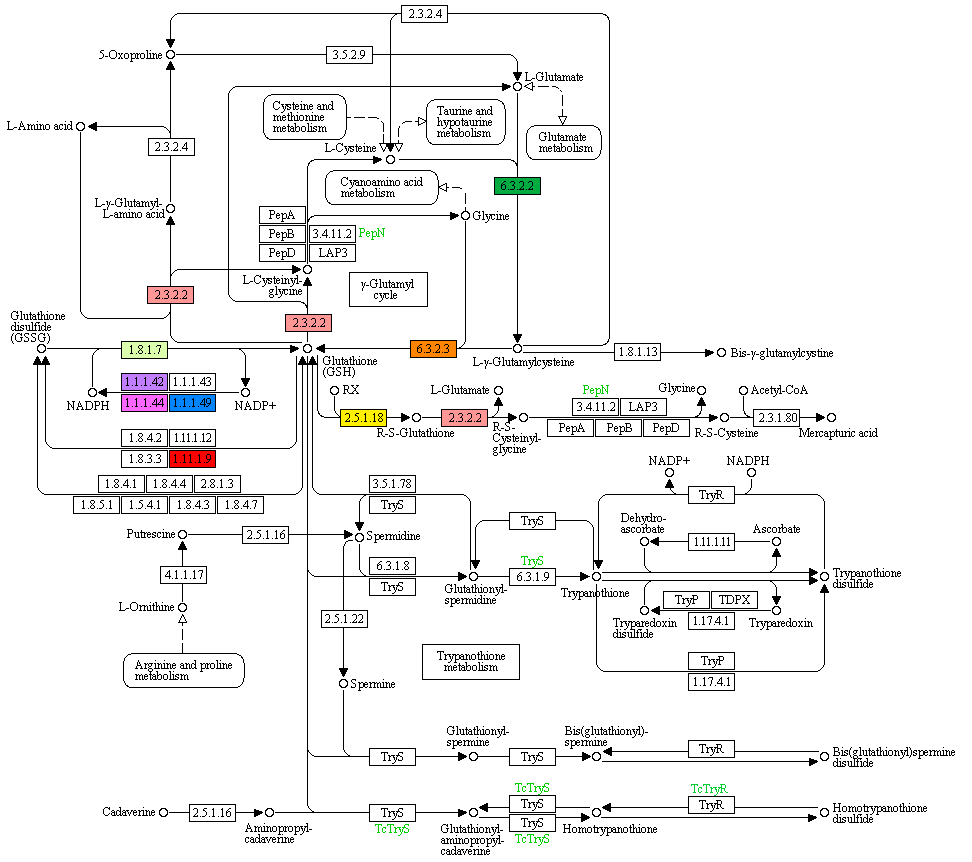


Note: The enzymes encoded by transcripts that are present in the subtractive library are highlighted with color combinations, in which the same color represents the same product.

**Additional file 2: Figure S4**- Carbohydrate metabolism involved in the reorganization of the plant cell wall during organogenesis of the nodule (ID Kegg-map00500).


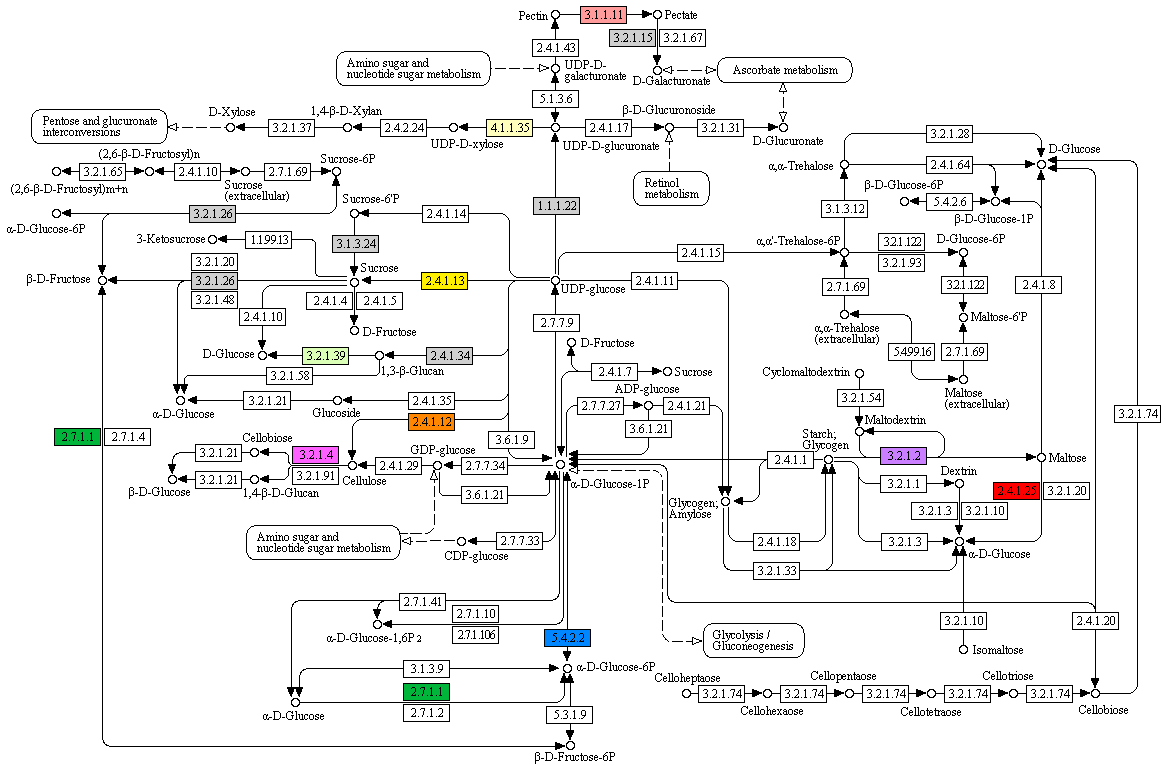


Note: The enzymes encoded by transcripts that are present in the subtractive library are highlighted with color combinations, in which the same color represents the same product.

**Additional file 2: Figure S5.** Spots of whole-cell protein extracts of soybean roots inoculated (left images) and non-inoculated (right images) with *B. japonicum*. Proteins identified corresponds to sucrose synthase (1) and putative glutathione-S-transferase (2).


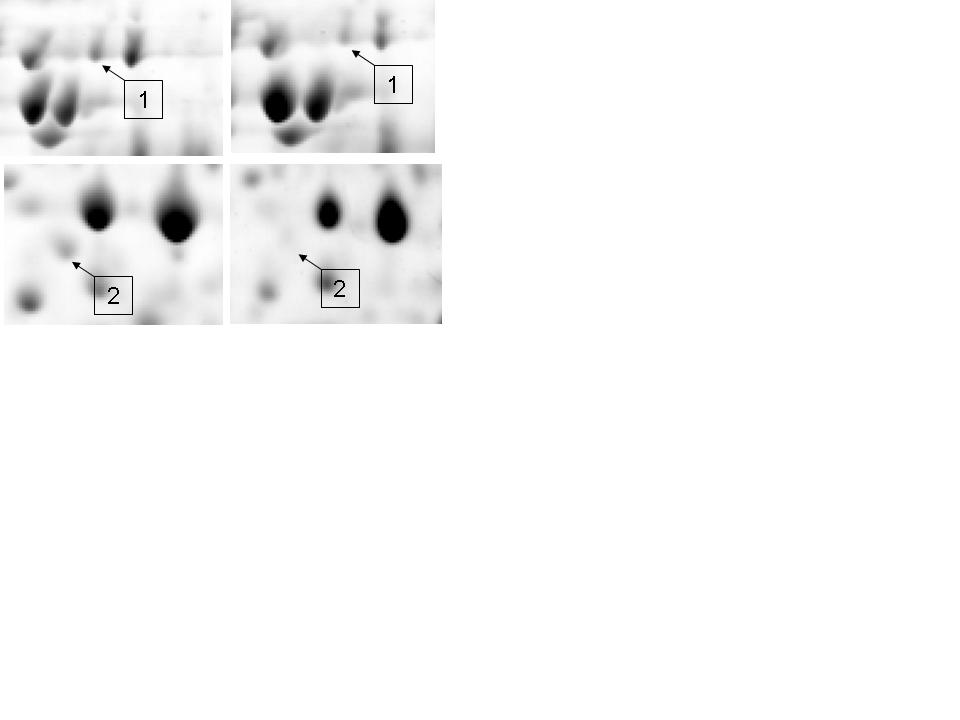

Supplement: Additional file 2: Figure S1 — Glycolysis pathway during nodulation of soybean cultivar Conquista at 10 days after inoculation with B. japonicum CPAC 15 (ID Kegg map00010). Figure S2. Krebs cycle of soybean cultivar Conquista at 10 days after inoculation with B. japonicum CPAC 15 (ID Kegg map00020). Figure S3. Glutathione metabolism – the antioxidant defense system present in the nodulation (ID Kegg- map00480). Figure S4. Carbohydrate metabolism involved in the reorganization of the plant cell wall during organogenesis of the nodule (ID Kegg-map00500). Figure S5. Spots of whole-cell protein extracts of soybean roots inoculated (left images) and non-inoculated (right images) with B. japonicum. Proteins identified corresponds to sucrose synthase (1) and putative glutathione-S-transferase (2). [file 1471-2164-14-153-S2.doc]
